# Comparison of Aerobic and Muscular Power Between Junior/U23 Slalom and Sprint Paddlers: An Analysis of International Medalists and Non-medalists

**DOI:** 10.3389/fphys.2020.617041

**Published:** 2021-01-20

**Authors:** Viktor Bielik, Leonard Lendvorský, Matej Vajda, Peter Lopata, Pavel Ružbarský, Ivan Gustavo Masselli dos Reis, Leonardo Henrique Dalcheco Messias

**Affiliations:** ^1^Department of Biological and Medical Sciences, Faculty of Physical Education and Sports, Comenius University, Bratislava, Slovakia; ^2^National Sport Center, Bratislava, Slovakia; ^3^Department of Sports Kinanthropology, Faculty of Sports, University of Prešov, Prešov, Slovakia; ^4^Laboratory of Multidisciplinary Research, Universidade São Francisco, Bragança Paulista, Brazil

**Keywords:** canoeing, performance, young athletes, maximal oxygen uptake, muscular power

## Abstract

This study aimed to compare the aerobic power (treadmill running) and muscle power (bench press and bench pull) of Junior/U23 paddlers from Slovakia who won medals in international championships with that of those who did not take the podium. Forty-three Slovak Junior/U23 paddlers (sprint = 24, medalists = 8, non-medalists = 16; slalom = 19, medalists = 11, non-medalists = 8) were tested in 2018 and 2019 after the world championships. The maximal oxygen uptake (VO_2max_) and the velocity at maximal oxygen uptake (vVO_2max_) were determined by the incremental running protocol (0% slope and 1 km⋅h^–1^ increments every minute until volitional exhaustion). Mean maximal power from the entire concentric phase was recorded during bench press and bench pull exercises by the validated TENDO weightlifting analyzer. No interaction was obtained between medal and canoe discipline for VO_2max_ (*p* = 0.069, *F* = 3.495), vVO_2max_ (*p* = 0.552, *F* = 0.361) and absolute (bench press: *p* = 0.486, *F* = 0.495; bench pull: *p* = 0.429, *F* = 0.640) or relative (bench press: *p* = 0.767, *F* = 0.089; bench pull: *p* = 0.696, *F* = 0.155) mean maximal power. Conversely, a significant effect for the medal on the bench press (absolute *p* = 0.017, *F* = 6.170; relative *p* = 0.043, *F* = 4.384) and the bench pull (absolute *p* = 0.041, *F* = 4.470) mean maximal power were observed. Our study indicates the absolute mean power on the bench press as a prerequisite for success in international Junior/U23 championships of slalom and sprint canoeing. However, the mean power on bench pull seems to have a deeper influence on sprint paddlers when compared to slalom athletes. Regarding the aerobic power, the data from the treadmill testing did not reveal outcomes between medalists and non-medalists. This result can be associated with the lack of specificity of the incremental treadmill testing for canoeing, and future studies are encouraged to propose specific protocols to compare the aerobic power of medalists and non-medalists in international slalom and sprint championships.

## Introduction

Canoe sprint and canoe slalom are sports that have been included in the summer Olympic Games since Berlin (1936) and Barcelona (1992), respectively (International Canoe Federation, 2020). Paddling technique along with high force development plays a crucial role for high performance on slalom (Zamparo et al., 2006; Messias et al., 2014, 2015, 2018; Baláš et al., 2020) and sprint (Michael et al., 2008; Pickett et al., 2018) canoeing. Studies also suggest an inverse association between aerobic metabolism and race time for both (Borges et al., 2015; Ferrari et al., 2017). Thus, strength and conditioning coaches must plan strategies to improve aerobic fitness and muscle strength/power of paddlers (García-Pallarés and Izquierdo, 2011; Rawlley-Singh and King, 2020). Such development, however, should also consider Junior and U23 athletes. In this sense, Bielik et al. (2018) demonstrates that the aerobic and muscle power of Slovak Junior/U23 paddlers who took the podium in international canoe sprint championships from 1995 to 2016 were ∼10% higher than the non-medalists. However, it remains to be demonstrated if a similar outcome occurs for slalom athletes.

Although canoe sprint considers linear courses in flat water, slalom paddlers must negotiate suspended poles in whitewater courses avoiding penalty (International Canoe Federation, 2020). Slalom gates are mounted on artificial or natural courses and vary substantially among competitions (Messias et al., 2014). The average race duration in official slalom courses ranges from 90 to 120 s and is dependent on the length of course, the difficulty of the rapids, and the number of gates (Nibali et al., 2011). Regarding sprint canoe, the race time varies substantially among the distances challenged in Olympic games (i.e., individual boats for male athletes: 200 m = 34–35 s; 1000 m = 205–215 s) (Borges et al., 2015). Additionally, race-to-race slalom variability of 1.2–2.1% (Nibali et al., 2011) is greater than on canoe sprint (0.7–1.5%) (Bonetti and Hopkins, 2010). These marked differences between canoeing disciplines should be taken into account when prescribing training sessions. To this end, strength, and conditioning coaches must understand if aerobic and muscular power are discriminating factors for successful attainment in international competitions. Moreover, this perspective needs to be extended in terms of canoeing disciplines (i.e., sprint vs. slalom) for proper training planning.

Therefore, we advance this issue by comparing aerobic power (treadmill running) and muscle power (bench press and bench pull) of Junior/U23 paddlers from Slovakia who won medals in international championships with that of those who did not take the podium. Slovakia has collected impressive results over the years in both canoeing disciplines, and their athlete development program is recognized worldwide. Although paddling techniques and physiological demands may vary between sprint and slalom paddlers (McKenzie and Berglund, 2019), evidence suggests the aerobic and muscle mean power as prerequisites for high performance in both disciplines in senior athletes (Bielik et al., 2019). However, young kayakers do not train on ergometers and, thus, are not fully accustomed to paddling out of the water. Otherwise, running is a common training routine in canoeing, especially at younger ages. Thus, we hypothesized that the physical fitness variables of Slovak Junior/U23 medalists to be higher than non-medalists but without significant effect in terms of canoeing discipline.

## Materials and Methods

### Design

This is an experimental and cross-sectional study comparing the aerobic power and muscle mean power of Junior/U23 Slovak medalist and non-medalist paddlers from sprint and slalom disciplines. Gold, silver, or bronze medals were won at the 2018 and 2019 Junior/U23 European and World championships. The ICF Junior/U23 Canoe Slalom European and World Championships occurred at the end of July in both years (2018: Ivrea, Italy, July 17–22; 2019: Krakow, Poland, July 16–21). Likewise, the ICF Junior/U23 Canoe Sprint European and World Championships occurred practically at the same period during 2018 (Plovdiv, Bulgaria, July 26–29) and 2019 (Piteşti, Romania, August 1–4). The athletes were evaluated in the same period after these championships as a part of the medical examination after the season is over in September of each year.

The evaluations occurred at the National Sport Center of Slovakia. Procedures followed standard testing protocols used for canoeing at the National Sport Center since 2004 (Bielik et al., 2018, 2019). Aerobic power and muscle mean power measurements were performed under laboratory conditions (18–22°C, 45–55% relative humidity). Researchers instructed athletes to avoid any vigorous exercise 48 h before the testing. The athletes were also instructed to follow their usual diet. Aerobic and muscle power measurements were carried out after an overnight fast and standardized breakfast before the examination. Body fat assessment was performed by the segmental multifrequency bioelectrical impedance analysis InBody_770_ (BioSpace, Los Angeles, CA, United States).

Our sample consisted of athletes from various clubs and/or training groups. Thus, the training structure over the years may differ due to seasonal, climatic, discipline specificity, and financial conditions. Because not all athletes kept their training diary, the description of a generalized training structure would be confusing. On the other hand, all training programs were annually designed and approved by the national federation.

### Subjects

Our sample consisted of 43 male Slovak Junior/U23 paddlers (slalom = 19, sprint = 24). Regarding sprint athletes, eight were medalists at the European and World Championships and 16 did not achieve the podium. Concerning slalom athletes, 11 won medals at the European and World Championships, and eight were non-medalists. Paddlers signed informed consent before the tests. For athletes under 18 years old, informed consent was also obtained from their parents. All experimental procedures were approved by the Ethics Committee of the Faculty of Physical Education and Sports of Comenius University and were conducted according to the ethical standards of Helsinki.

### Muscular Power Measurements

We acknowledge that not all participants were willing to lift maximal loads during the testing. Due to worries about possible injuries and fear from resulting interruptions in the training process, we examined the mean maximal power rather than the exact maximal strength of one maximal repetition (1RM). Mean maximal power from the entire concentric phase was recorded during bench press and bench pull by the validated TENDO Weightlifting Analyzer (Tendo Sport, Power Analyzer V-316, Trenèín, Slovakia) (Garnacho-Castaño et al., 2015; Goldsmith et al., 2019; Gonzalez et al., 2019). The TENDO Weightlifting Analyzer appliance attaches to conventional resistance-training equipment to measure the velocity of movement from which muscle power is calculated. It was placed on the floor and attached via its nylon cord to the Olympic barbell onto which the weights were loaded.

Data related to the average power of the concentric phase during bench press and bench pull were recorded and calculated in the computer. The Tendo Weightlifting Analyzer System emerged as a reliable system for measuring movement velocity and estimating power in resistance exercises (Garnacho-Castaño et al., 2015). The validity and reliability of Tendo Weightlifting Analyzer Systems were established by Jennings et al. (2005) and Goldsmith et al. (2019). The system consists of a velocity sensor connected to the load by a Kevlar cable that, through an interface, instantly transmits the vertical velocity of the bar to specific software (Tendo Weight-lifting Analyzer 3.0.4). The velocity sensor comprises an optical sensor and a light source within a slotted disk to control the movement and time of the measurement as well as a continuous current motor for the orientation of the movement. The system measures vertical average and peak velocity of the weight lifted. Using the known mass, the system calculates the average power in the concentric phase. A cable surrounding the slotted disk is connected to the bar or the corresponding load using Velcro. When the disk turns, the light shines from the slotted disk, converting it into electric impulses read by the optic sensor. In weight exercises, muscles lift a mass m against the gravity acceleration g by applying a force F (Equation 1) at a velocity v, producing average power P (Equation 2).

(1) F=m×g

(2) P=F×v

Calibration was performed according to the manufacturer’s guidelines before each measurement. The warm-up consisted of 5 min of general warm-up followed by 1 series of a given exercise (bench press or bench pull) with an empty bar (20 kg). Subjects performed trials of three repetitions on each load with maximal effort with a 3-min rest period interval. The testing protocol started with an external load of 20 kg (i.e., Olympic barbell only) and gradually increased by 10 or 5 kg. The test was terminated upon reaching a plateau or decrease in maximal mean power output. Repetitions began with a controlled lowering of the weight, immediately followed by a straight explosive movement upward with emphasis to perform the concentric phase as quickly as possible. Only the repetition with the highest power output at each load was considered for subsequent analysis.

### Incremental Running Test for Aerobic Power Determination

Slovak paddlers performed an incremental running protocol for maximal oxygen uptake (VO_2max_) and velocity at maximal oxygen uptake (vVO_2max_). After familiarization with the ergometer, athletes completed a maximal incremental running test on a motorized treadmill (h/p/cosmos Sports & Medical GmbH, h/p/cosmos Pulsar, Nußdorf, Germany). The warm-up consisted of 5 min of easy running and dynamic stretching. The test started with 8 km⋅h^–1^, and the velocity was increased by 1 km⋅h^–1^ every minute until volitional exhaustion. A 0% slope was considered throughout the incremental test, and the last velocity increment was held for an entire minute. The test ended before voluntary exhaustion only if the oxygen uptake values leveled off or decreased despite increasing workload and ventilation and the respiratory exchange ratio was higher than 1.10. Researchers encouraged Slovak paddlers mostly during the last minute of the test. Respiratory variables were continuously measured by a breath-by-breath gas analyzer system power Cube (Ganshorn Medizin Electronic GmbH, PowerCube Ergo CSO2, Niederlauer, Germany). Equipment was calibrated before each session according to the manufacturer’s guidelines. At least two exercise physiologists supervised the procedures during the entire period. Oxygen uptake data collected during the last 10 s of each 1-min stage was averaged.

### Statistical Analyses

Data were compared on STATISTICA 7.0 (StatSoft, OK, United States) and expressed as mean and standard deviation (SD). Shapiro–Wilk and Levene tests confirmed the data’s normality and homogeneity, respectively. Two-way ANOVA considered medalists and canoeing discipline as independent variables and aerobic/mean muscular power as a dependent. Partial eta squared evaluated the effect size of two-way ANOVA following these criteria: small = 0.01, medium = 0.06, large = 0.14. In all cases, statistical significance was set at *P <* 0.05.

Based on the muscular power measurements, we performed an objective analysis on each axis of graph plots for evaluating data distribution and finding the spot where the prevalence of medal winners is higher for bench press power, bench pull power, and bench press–bench pull ratio. The latter variable was created to show the proportion between powers in the press and pull ergometers. The data range of each variable was calculated by the difference between the maximum and minimum values and a window of 25% data range was positioned at the respective axis coordinates to match the area at which the number of medal winners is the highest. If there were two or more areas with the same absolute number of medal winners, the window was positioned at the one closer to the majority of medal winners left out (the more central among outside medal winners).

## Results

The physical characteristics of the Slovak paddlers are shown in Table 1. The body composition was similar regardless of the canoeing discipline and medal. However, the slalom and sprint medalists were older than non-medalists (*p* = 0.002, *F* = 11.461, η^2^ = 0.227). Moreover, sprint paddlers were heavier (*p* = 0.001, *F* = 14.057, η^2^ = 0.265) and taller (*p* = 0.001, *F* = 12.350, η^2^ = 0.241) than slalom athletes regardless of the medal.

**TABLE 1:** Physical characteristics of the sprint and slalom Slovak paddlers.

***N* = 43**	**M.CSla (*n* = 11)**	**NM.CSla (*n* = 8)**	**M.CSpr (*n* = 8)**	**NM.CSpr (*n* = 16)**	***F***	***P***
Age (yrs)	18 ± 1	19 ± 1	19 ± 2	18 ± 1	0.056	0.813
Body mass (kg)	75.4 ± 4.6	73.6 ± 6.3	85.6 ± 8.6	79.9 ± 7.2	2.960	0.093
Height (cm)	176 ± 4	179 ± 4	186 ± 10	183 ± 5	0.095	0.759
Fat (%)	12.9 ± 2.3	8.9 ± 3.0	8.8 ± 2.2	9.8 ± 3.5	1.068	0.308

*M.CSla, canoe slalom medalists; NM.CSla, canoe slalom non-medalists; M.CSla, sprint medalists; NM.CSla, sprint non-medalists; p, *p*-value from ANOVA two-way; F, *F* value from ANOVA two-way; *P* < 0.05.*

**FIGURE 1:**
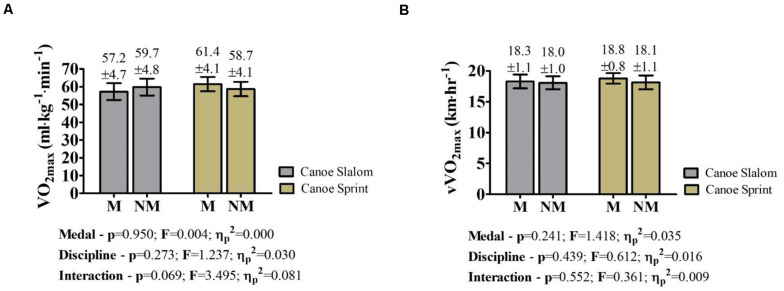
Comparison between Slovak medalists and non-medalists Junior/U23 sprint and slalom paddlers in terms of aerobic power; **(A)** Maximal oxygen uptake (VO_2_max); **(B)** Velocity at maximal oxygen uptake (vVO_2_max); M, medalists on European and World Championships; NM, non-medalists on European and World Championships; M.CSla, canoe slalom paddlers medalists; NM.CSla, canoe slalom paddlers non-medalists; M.CSla, sprint paddlers medalists; NM.CSla, sprint paddlers non-medalists; p, *p* value from ANOVA two-way; F, *F* value from ANOVA two-way; η_*P*_^2^ = Partial Eta Squared from ANOVA two-way; *P* < 0.05.

**FIGURE 2:**
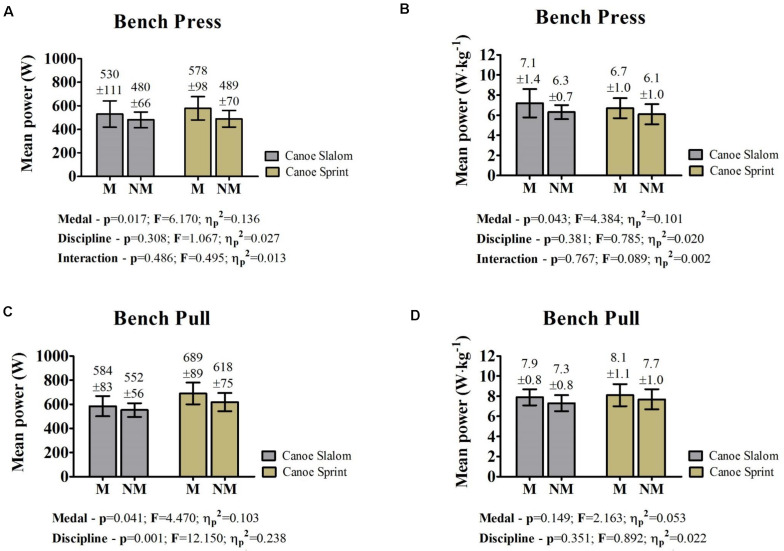
Comparison between Slovak medalists and non-medalists Junior/U23 sprint and slalom paddlers in terms of muscular power; **(A)** Absolute mean power developed on bench press; **(B)** Relative mean power developed on bench press; **(C)** Absolute mean power developed on bench pull; **(D)** Relative mean power developed on bench pull; M, medalists on European and World Championships; NM, non-medalists on European and World Championships; M.CSla, canoe slalom paddlers medalists; NM.CSla, canoe slalom paddlers non-medalists; M.CSla, sprint paddlers medalists; NM.CSla, sprint paddlers non-medalists; p, *p* value from ANOVA two-way; F, *F* value from ANOVA two-way; η_*p*_^2^ = Partial Eta Squared from ANOVA two-way; *P* < 0.05.

Junior/U23 sprint and slalom Slovak paddlers who took the podium at the European and World Championships had similar aerobic power to those non-medalists (Figure 1). No interaction between medal and canoe discipline was observed for the absolute and relative mean power on the bench press and pull (Figure 2). On the other hand, ANOVA showed that the mean power on the bench press of medalists was higher than non-medalists (Figures 2A,B). Additionally, significant effects for medal and discipline were pointed for the absolute mean power on bench pull (Figure 2C). Conversely, these differences did not occur on the relative values (Figure 2D).

**FIGURE 3:**
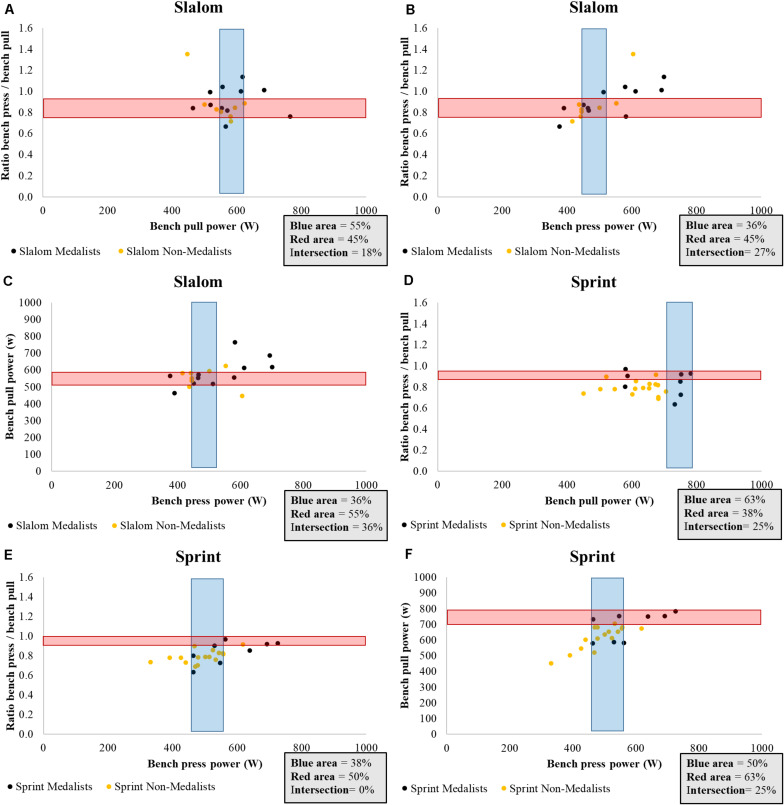
An objective analysis of the absolute mean power obtained from bench press and bench pull measurements by medalists and non-medalists in international Junior/U23 slalom and sprint canoeing championships; **(A)** Ratio of the bench pull and press mean power plotted against mean power developed by slalom paddlers on bench pull; **(B)** Ratio of the bench pull and press mean power plotted against mean power developed by slalom paddlers on the bench press; **(C)** Plot of the mean power on bench pull and bench press of slalom paddlers; **(D)** Ratio of the bench pull and press mean power plotted against mean power developed by sprint paddlers on bench pull; **(E)** Ratio of the bench pull and press mean power plotted against mean power developed by sprint paddlers on the bench press; **(F)** Plot of the mean power on bench pull and bench press of sprint paddlers; Red area refers to the *y*-axis; Blue area refers to the *x*-axis.

**FIGURE 4:**
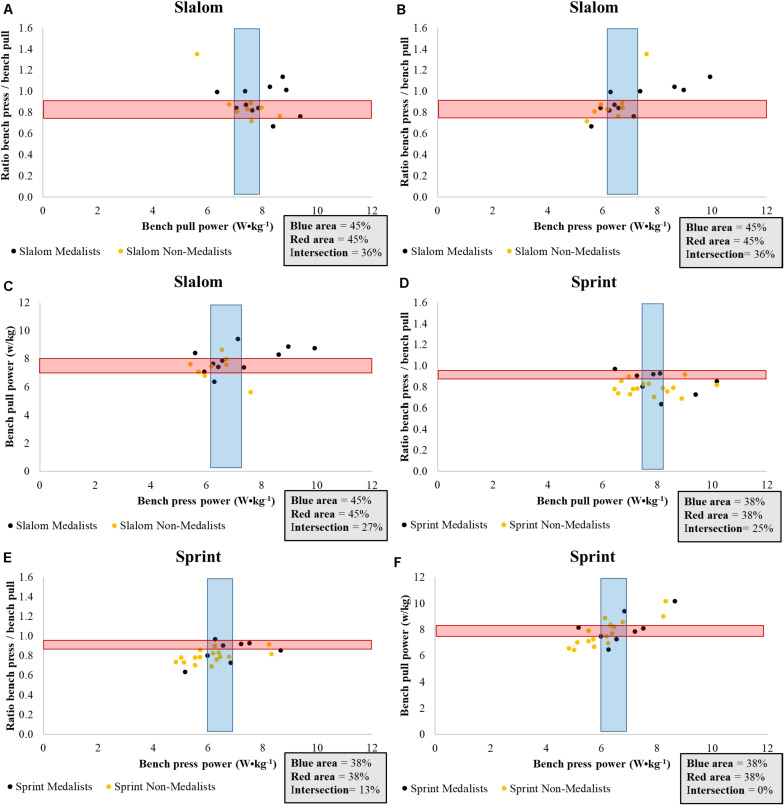
An objective analysis of the relative mean power obtained from bench press and bench pull measurements by medalists and non-medalists in international Junior/U23 slalom and sprint canoeing championships; **(A)** Ratio of the bench pull and press mean power plotted against mean power developed by slalom paddlers on bench pull; **(B)** Ratio of the bench pull and press mean power plotted against mean power developed by slalom paddlers on the bench press; **(C)** Plot of the mean power on bench pull and bench press of slalom paddlers; **(D)** Ratio of the bench pull and press mean power plotted against mean power developed by sprint paddlers on bench pull; **(E)** Ratio of the bench pull and press mean power plotted against mean power developed by sprint paddlers on the bench press; **(F)** Plot of the mean power on bench pull and bench press of sprint paddlers; Red area refers to the *y*-axis; Blue area refers to the *x*-axis.

The mean power on the bench press and bench pull of slalom paddlers were similar (medalists absolute mean power: *p* = 0.061, medalists relative mean power: *p* = 0.062, non-medalists absolute mean power: *p* = 0.079, non-medalists relative mean power: *p* = 0.069). Nevertheless, the mean power on the bench pull was consistently higher among sprint paddlers (medalists absolute mean power: *p* = 0.000, medalists relative mean power: *p* = 0.007, non-medalists absolute mean power: *p* = 0.000, non-medalists relative mean power: *p* = 0.000). The objective analysis between the mean power on the bench press and pull is shown in Figures 3, 4. In summary, less than half of the medalists were located at the intersection regardless of the analyses. Restrictedly to sprint paddlers, more than half of the medalists presented a higher absolute power on bench pull than the remaining athletes (Figure 3D). On the other hand, this scenario disappears in terms of relative power (Figure 4D).

## Discussion

This is the first study to compare the aerobic and muscular power of slalom and sprint paddler medalists and non-medalists in Junior/U23 Championships. Despite the similarity for aerobic power, it must be noted that these values are higher than those of merely active individuals. Restricted to the other outcomes, our hypothesis is only accepted for mean power on the bench press. Although the absolute mean power on the bench pull of sprint paddlers was higher compared to slalom paddlers, this difference disappears in terms of relative values. Therefore, the higher absolute mean power on the bench pull seems to be more related to anthropometric characteristics (i.e., body mass) than an exclusive prerequisite for sprint paddlers. On the other hand, both medalists and non-medalists in international sprint championships had higher mean power on bench pull when compared with the bench press, and this has deep implications for strength and conditioning training prescription.

### The Role of Aerobic Power for Junior/U23 Sprint and Slalom Paddlers

Bielik et al. (2018) suggests that aerobic power is not a guarantee, but a prerequisite for success in Junior/U23 International Sprint Championships. Sprint paddlers cover linear distances that vary from 200 to 5000 m, and metabolic contribution is different according to the trial (Byrnes and Kearney, 1997). Conversely, the whitewater courses require several moments of acceleration and deceleration (Messias et al., 2014), and the aerobic and anaerobic energy systems contribute almost equally (Zamparo et al., 2006). Data reported in Figure 1 confirm previous suggestions regarding the oxidative relevance for sprint and slalom paddlers (Michael et al., 2008; Borges et al., 2015; Ferrari et al., 2017). Likewise, Bielik et al. (2019) show that Olympic slalom medalists had similar VO_2max_ and vVO_2max_ than the non-medalists. Although ANOVA did not point out that aerobic power delimitates the chance of taking the podium in Junior/U23 slalom and sprint championships, the interaction of discipline and medal nearly reached significance for VO_2max_ (Figure 1A). Taken together, our results have two main inferences. To begin with, VO_2max_ is more likely to affect performance on sprint canoe than on slalom, but this requires further experimentation. On the other hand, it is important to state that, although aerobic power may not directly influence the success at the International Junior/U23 Slalom Championships, both medalists and non-medalists had higher values of VO_2max_ when compared to healthy subjects (Edvardsen et al., 2013).

It is valid to state that aerobic power was measured without the canoeing specificity (i.e., treadmill running), which is a limitation of this study. Previous reports suggested ergometers to access aerobic fitness with more ecologically valid settings (Manchado-Gobatto et al., 2014; Ferrari et al., 2017). However, some of these require unique tools or several days of testing, which may hamper its application in a daily routine. Therefore, further studies are encouraged to propose protocols with great canoeing specificity and practical application to determine aerobic power. Meanwhile, the aerobic power from treadmill running can offer insights for both canoeing disciplines, but coaches must be aware that such measurement lacks the canoeing specificity and can be protocol-dependent (Bielik et al., 2019).

### The Role of Muscle Power for Junior/U23 Sprint and Slalom Paddlers

Trunk and upper extremities hugely participate in paddling efforts to overcome water resistance on both sprint and slalom races (Messias et al., 2014; García-García et al., 2015). Thus, strength and conditioning coaches regularly prescribe dry-land training based on the bench press and pull for paddlers (García-Pallarés et al., 2009; Bielik et al., 2018). This factor explains why these exercises are adopted for studying the strength/power of these athletes (García-Pallarés et al., 2009; García-Pallarés and Izquierdo, 2011; Ualí et al., 2012; McKean and Burkett, 2014; Bielik et al., 2018).

The fact that sprint athletes are heavier (regardless of the medal factor) than those from slalom can explain why the absolute mean power on bench pull of these athletes was higher. However, because sprint and slalom paddlers had similar relative mean power on the bench pull, we suggest the relevance of this parameter for both disciplines. Curiously, the mean power (absolute and relative) on the bench press and bench pull of sprint paddlers were not proportional. Sprint paddlers cover a linear flat-water course repeating the same pulling and pushing cycle during forwarding strokes. However, it is unclear to what extent the proportion of the power developed during the press and pull measurements affect the performance in sprint events. Such limitation is also visualized for canoe slalom.

Thus, the main outcome of the intersection presented in Figures 3, 4 is referred to as the necessity of maintaining a good proportion (i.e., 1.0) between mean power on the bench press and pull while isolated improvements in these parameters are acquired. For neither slalom nor sprint paddlers does this seem to be the case. Restricted to slalom paddlers, more than half of medalists were located in the blue area in terms of absolute mean power on bench pull (∼570–615 w) (Figure 3A). Such a result, on the other hand, did not occur for bench press (Figure 3B). Therefore, it is safe to suggest that the mean power on the bench pull seems to be associated with the performance in international Junior/U23 Slalom Championships. On the other hand, more than half of non-medalists (orange points) were also located in this area, suggesting that other factors such as paddling technique and the ability to predict the natural and artificial obstacles, also affect the performance in slalom trials.

Regarding the sprint paddlers, Figure 3D probably demonstrates the most interesting result of this study. In terms of absolute power, more than half of sprint medalists were located in the blue area (∼700–790 w). This indicates that improvements in absolute mean power on the bench pull seems to be an important physical prerequisite for success in international Junior/U23 sprint championships. However, the same perspective cannot be valid for the relative mean power on bench pull (Figure 4D). Therefore, although the relative mean power enables further comparisons between paddlers with distinct body mass, the absolute mean power on the bench pull seems to be a better indicator of success in international Junior/U23 Sprint Championships. We cannot assume that high absolute mean power levels in bench pull will lead athletes to the podium because three sprint medalists were outside the blue area, but certainly their chances will increase. Last, once the flexion of the elbow and shoulder is extensively used in rowing, bench pull is commonly suggested as a specific evaluation for these athletes (Lawton et al., 2011). The same context seems to occur for Junior/U23 sprint paddlers.

### Limitations and Practical Applications

Regarding the limitations, the paddlers were not separated according to their specialty (e.g., distances) or even equipment (e.g., boats, paddles) and other (e.g., individual or team challenges) characteristics. Moreover, our results cannot predict performance on sprint and slalom disciplines. Studies have deeply discussed the role of other components, such as paddling technique (Hunter et al., 2008; Hunter, 2009), psychological factors (Hill et al., 2010), and cognitive enhancement (MacIntyre and Moran, 2007) for these athletes. Future studies are encouraged to associate these components with the outcomes presented in this investigation. The results presented in this study are valid for mean power on the bench press and pull. Further investigations should answer if similar outcomes are transposed to the peak power. Last, we retake that the aerobic power was measured without the canoeing specificity, which is also a limitation of this study.

Concerning the practical applications, our results provide important insights for strength and conditioning coaches. First, we take back previous suggestions that aerobic power is an important component for high performance on slalom and sprint canoe. Therefore, some training sessions must be conducted to improve the aerobic metabolism. Furthermore, absolute and relative mean power on the bench press is a measurement that discriminates Junior/U23 medalists and non-medalists in both canoeing disciplines. On the other hand, the absolute mean power on the bench pull seems to be a better measurement for sprint paddlers when compared to slalom. Thus, strength and conditioning coaches must be aware of this information for both evaluation and training purposes.

## Conclusion

In summary, our study indicates the absolute mean power on the bench press as a prerequisite for success in International Junior/U23 Championships of slalom and sprint canoeing. However, the mean power on the bench pull seems to have a deeper influence on sprint paddlers when compared to slalom athletes. Regarding the aerobic power, further studies are encouraged to validate protocols for measuring this parameter with great canoeing specific and practical application. Although previous reports have suggested that aerobic metabolism plays an important role in slalom and sprint performance, the data from the treadmill testing did not reveal outcomes between medalists and non-medalists.

## Data Availability Statement

The raw data supporting the conclusions of this article will be made available by the authors, without undue reservation.

## Ethics Statement

The studies involving human participants were reviewed and approved by the Faculty of Physical Education and Sports of the Comenius University. Written informed consent to participate in this study was provided by the participants’ legal guardian/next of kin.

## Author Contributions

VB, LL, MV, PL, and PR contributed to experimental design application, acquisition and data analysis, the writing of the main manuscript text, proposal of ideas, the conception, design of the work, and interpretation of data. IM and LM contributed to data analysis, the writing of the main manuscript text, interpretation of data, and preparing the figures and table. All authors reviewed the manuscript and approved the submitted version.

## Conflict of Interest

The authors declare that the research was conducted in the absence of any commercial or financial relationships that could be construed as a potential conflict of interest.
